# BMP9, but not BMP10, acts as a quiescence factor on tumor growth, vessel normalization and metastasis in a mouse model of breast cancer

**DOI:** 10.1186/s13046-018-0885-1

**Published:** 2018-08-30

**Authors:** Marie Ouarné, Claire Bouvard, Gabriela Boneva, Christine Mallet, Johnny Ribeiro, Agnès Desroches-Castan, Emmanuelle Soleilhac, Emmanuelle Tillet, Olivier Peyruchaud, Sabine Bailly

**Affiliations:** 1 Univ. Grenoble Alpes, Inserm, CEA, BIG-Biologie du Cancer et de l’Infection, 38000 Grenoble, France; 2 Inserm, U1033, Lyon, France; 3 Université Claude Bernard Lyon 1, Villeurbanne, France; 4 Faculté de Médecine de Lyon Est, Lyon, France; 5 Univ. Grenoble Alpes, Inserm, CEA, BIG-Biologie à Grande Echelle, 38000 Grenoble, France

**Keywords:** BMP9, BMP10, Tumor angiogenesis, E0771, Mammary tumor, Metastasis

## Abstract

**Background:**

Angiogenesis has become an attractive target for cancer therapy. However, despite the initial success of anti-VEGF (Vascular endothelial growth factor) therapies, the overall survival appears only modestly improved and resistance to therapy often develops. Other anti-angiogenic targets are thus urgently needed. The predominant expression of the type I BMP (bone morphogenetic protein) receptor ALK1 (activin receptor-like kinase 1) in endothelial cells makes it an attractive target, and phase I/II trials are currently being conducted. ALK1 binds with strong affinity to two ligands that belong to the TGF-ß family, BMP9 and BMP10. In the present work, we addressed their specific roles in tumor angiogenesis, cancer development and metastasis in a mammary cancer model.

**Methods:**

For this, we used knockout (KO) mice for BMP9 (constitutive *Gdf2-deficient*), for BMP10 (inducible *Bmp10-deficient*) and double KO mice (*Gdf2* and *Bmp10*) in a syngeneic immunocompetent orthotopic mouse model of spontaneous metastatic breast cancer (E0771).

**Results:**

Our studies demonstrate a specific role for BMP9 in the E0771 mammary carcinoma model. *Gdf2* deletion increased tumor growth while inhibiting vessel maturation and tumor perfusion. *Gdf2* deletion also increased the number and the mean size of lung metastases. On the other hand, *Bmp10* deletion did not significantly affect the E0771 mammary model and the double deletion (*Gdf2* and *Bmp10*) did not lead to a stronger phenotype than the single *Gdf2* deletion.

**Conclusions:**

Altogether, our data show that in a tumor environment BMP9 and BMP10 play different roles and thus blocking their shared receptor ALK1 is maybe not appropriate. Indeed, BMP9, but not BMP10, acts as a quiescence factor on tumor growth, lung metastasis and vessel normalization. Our results also support that activating rather than blocking the BMP9 pathway could be a new strategy for tumor vessel normalization in order to treat breast cancer.

**Electronic supplementary material:**

The online version of this article (10.1186/s13046-018-0885-1) contains supplementary material, which is available to authorized users.

## Background

Neovascularization is one of the hallmarks of cancer as it is a necessary process to provide tumor with its metabolic requirements, while simultaneously creating an escape route by which cancer cells will disseminate [1, 2]. Thus, several angiogenesis inhibitors have been developed and most of them target vascular endothelial growth factor (VEGF) signaling [3]. Today, VEGF inhibitors are included in first-line therapies against advanced and metastatic cancers [4]. However, the lack of substantial improvements of overall survival and resistance issues clearly support the crucial need for identification of alternative and/or complementary targets for drug development [5].

The transforming growth factor (TGF)-β family type I receptor ALK1 (activin receptor-like kinase 1), which is mainly expressed on endothelial cells, has been identified as a potential target for anti-angiogenic cancer treatment [6, 7]. ALK1 is indeed an essential receptor in vascular development, as genetic ablation of *Acvrl1* (encoding ALK1) in mice results in embryonic lethality due to vasculogenic or angiogenic defects [8, 9]. In addition, mutations of the genes *ACVRL1* and *ENG,* encoding the co-receptor endoglin, are responsible of the Rendu-Osler syndrome also known as hereditary hemorrhagic telangiectasia (HHT) [10, 11]. The discovery of the high affinity binding of BMP9 (bone morphogenetic protein) and BMP10 to ALK1 has revealed the key role of these two ligands in vascular development [12, 13]. BMP9 and BMP10 are very similar at the amino-acids levels but they differ in several ways. First, the site of expression is different, as BMP9 is mainly produced by the liver [14] while BMP10 is mostly produced by the heart [15], although they are both detected in blood [16, 17]. Second, although BMP9 and BMP10 both bind to the type I receptor ALK1 with high affinity, only BMP9 binds to ALK2 [18] and their affinities for the type II receptors differ [19]. Knockout mice for *Gdf2* (encoding BMP9) are viable and fertile with no overt defect in blood vessel development [20] while *Bmp10*^−/−^ mice die during embryonic development due to heart defects [21]. Still, we could show that *Gdf2*-deficient mice present defects in lymphatic valve formation and lymph drainage, supporting a specific role for BMP9 in lymphatic maturation [22]. Moreover, it was recently shown that BMP9 and BMP10 play redundant roles in retinal vascularization and ductus arteriosus closure [17, 20, 23]. Together, these data clearly demonstrate, in vivo, the crucial roles of ALK1 and its two ligands, BMP9 and BMP10, in vascular development. However, their precise role during the complex process of physiological angiogenesis has proven difficult to pinpoint from in vitro studies, as their actions appear highly concentration- and context-dependent [24].

Still, due to the key role of this pathway in angiogenesis, ALK1 has been identified as an interesting target in tumor angiogenesis [6, 25]. Pharmacological targeting of ALK1 has been evaluated using either a neutralizing anti-ALK1 antibody (PF-03446962) or a soluble form of ALK1 (Dalantercept) that will trap the biological ligands of ALK1, BMP9 and BMP10 [26, 27]. Most of the published preclinical studies, using these neutralizing tools, reported a decrease in tumor volume, tumor angiogenesis and metastasis [28–30]. Thus, several phase I/II studies using these agents are currently being conducted. However, despite being generally well tolerated, no efficacy of ALK1 blockade has been demonstrated to date [5, 25, 31, 32].

Little is known about the respective roles of BMP9 and BMP10 in tumor angiogenesis, cancer development and metastatic dissemination. BMP9 has been shown to play, via ALK2, a direct role on tumor growth as an autocrine growth factor in hepatocarcinoma [33]. The roles of BMP9 and BMP10 have also been studied in different cancers using tumor cells overexpressing either BMP9 or BMP10 [34–37], although BMP9 and BMP10 are not or moderately expressed in most of the tumors that have been studied so far. In these studies, BMP9 and BMP10 were described as tumor suppressors acting directly on cancer cells but their roles in tumor angiogenesis have not been investigated.

Herein, we studied the respective roles of BMP9 and BMP10 in tumor growth, tumor angiogenesis and metastatic dissemination using the murine syngeneic orthotopic mammary cancer model (E0771) [38, 39]. To understand the contribution of BMP9 and BMP10 ligands, we made use of *Gdf2*-deficient mice, inducible *Bmp10-*deficient mice and double *Gdf2- and Bmp10-*deficient mice. Our study demonstrates a specific role for BMP9 in tumor growth, tumor angiogenesis and lung metastasis in the E0771 model. *Bmp10* deletion did not significantly affect this mammary model, and the double deletion did not lead to a stronger phenotype than the single deletion of *Gdf2*.

## Methods

### Cell lines

E0771 (Tebu-Bio) breast cancer mouse cells were maintained in culture in RPMI-1640 (Life Technologies) supplemented with 10% fetal calf serum (FCS) and were used below passage 5. Mouse endothelial cells H5V (gift from Dr. A. Mantovani) were maintained in culture in DMEM 4.5 g/L glucose (Life Technologies) supplemented with 10% FCS. Breast epithelial cells EpH4 (Clone J3B1A, a gift from Dr. P. Soulie) were maintained in culture in DMEM/F12 (Life Technologies) supplemented with 10% FCS. All cells were tested for Mycoplasma (MycoAlert™ PLUS, Lonza).

### *Gdf2*^−/−^, *Bmp10*-cKO and double-KO (*Gdf2*^*−/−*^*a*nd *Bmp10*) mice

*Gdf2*^**−/−**^mice generation was previously described [20]. To circumvent the early embryonic lethality of *Bmp10-*KO mice [21], the Institut Clinique de la Souris (Illkirch, France) generated for us a *Bmp10*^lox/lox^ mice by flanking loxP sites around exon2. These mice were then crossed with the Rosa26CreER^T2^ mice provided by Pr. P. Chambon (IGBMC, Illkirch, France) [40] to generate conditional knockout mice for *Bmp10* (*Bmp10*-cKO mice). Intraperitoneal injections of tamoxifen (1 mg) were performed for five days in 3-week-old control (*Bmp10*^lox/lox^) and *Bmp10*-cKO (Rosa26CreER^T2^;*Bmp10*^lox/lox^) in order to delete *Bmp10*. Rosa26CreER^T2^;*Bmp10*-cKO mice were crossed with *Gdf2*^**−/−**^ mice to generate *Gdf2*^**−/−**^*;Bmp10*^lox/lox^ that will be referred to as double-KO mice. The same protocol as for *Bmp10*-cKO mice was used to delete *Bmp10*. *Bmp10*-cKO mice were maintained in the C57BL/6 background. All mice described were viable and fertile.

### Orthotopic syngeneic mammary tumor models

For the E0771 model, 10^5^ cells were injected orthotopically into the fourth mammary fat pad of isofluran-anesthesized C57BL/6 CTL and KO females at 6 weeks of age. Mice were euthanized at 9 weeks of age 10 min after intravenous injection of 50 μL of tomato lectin (DyLight 488 *Lycopersicon esculentum*, DL-1174, Vector Laboratories, 1 mg/mL). Tumor size was measured with calipers, and the volume was calculated according to the formula (L*w^2^)/2 where L and w stand for length and width respectively.

### Tissue preparation and immunostainings

Tumors were fixed in 4% paraformaldehyde over night at 4 °C and embedded in Tissue-Tek^R^ OCT™ compound (optimum cutting temperature) (Sakura) for frozen sections or in paraffin.

For immunohistochemistry, paraffin-embedded sections were deparaffinized and rehydrated followed by citrate antigen retrieval. Blocking was performed in 2% BSA in TBS. Sections were stained using antibodies to PCNA (dilution 1:6000; Abcam [PC10] Ab29) and active caspase-3 (dilution 1:1000; R&D Systems AF835). Appropriate biotinylated secondary antibodies were used (Vector Laboratories). Sections were incubated with an avidin-biotin complex (Vectastain ABC kit; Vector Laboratories) and staining revealed by addition of 3.3′-diaminobenzidine (Liquid DAB+ Substrate Chromogen System; Dako). Counterstaining was performed using hematoxylin or fast nuclear red. Images were acquired with a Zeiss Axioplan microscope and analyzed using Axiovision 4.8 software.

For immunofluorescence, frozen sections were fixed in 4% paraformaldehyde and permeabilized in 0.5% triton in PBS. Blocking was performed in 2% BSA in PBS. Sections were stained using antibodies to podocalyxin (dilution 1:50; R&D Systems AF1556), FITC (dilution 1:100; Alexa Fluor 488 conjugated; Jackson Immunoresearch Laboratories [1F8-1E4] 200–542-037), α-SMA (dilution 1:200; Cy3 conjugated; Sigma Aldrich [1A4] C6198) and LYVE-1 (dilution 1:100; R&D Systems MAB2125). Appropriate secondary antibodies conjugated with fluorochromes were used (Jackson Immunoresearch Laboratories). Apoptosis was analyzed by the indirect TUNEL (Terminal deoxynucleotidyl transferase dUTP nick end labeling) method (ApopTag® Red In Situ Apoptosis Detection Kit from Millipore). Nucleus were stained using Hoechst blue (33,342, Sigma). Images were acquired with a Zeiss ApoTome microscope, treated using Zen Blue software and analyzed using ImageJ software.

For necrosis quantification, paraffin-embedded sections were stained with hematoxylin and eosin. Images were acquired using AxioScan Z1 (Zeiss) slide scanner and analyzed using Zen Blue software.

All quantifications were performed by assessing 3 to 5 images per tumor using ImageJ software.

### Quantification of lung metastases

After being inflated using 4% paraformaldehyde, lungs were embedded in paraffin upon tissue fixation in 4% paraformaldehyde. The metastatic burden was assessed by serial sectioning of the entire lungs. Hematoxylin and eosin staining was performed on sections every 200 μm. Images were acquired using AxioScan Z1 (Zeiss) slide scanner and analyzed using Zen Blue software.

### Western blot analysis

Cells were stimulated in 0% FCS with recombinant BMP9 (R&D Systems) at the concentration indicated for 1 h after 1 h30 of serum deprivation. Cell extracts were lysed in 50 mmol/L Tris-HCl pH 7.4, 0.5 mol/L NaCl and a cocktail of protease inhibitors (Sigma) by sonication. 20 μg of proteins from cell lysates were separated on a SDS/PAGE, 4–20% (Bio-Rad) and analyzed by immunoblotting with anti-pSmad1/5/9 antibody (Cell Signaling #9511). The same membrane was reprobed with a monoclonal antibody against β-actin (clone AC-15; Sigma) to confirm equal protein loading.

### RT-PCR analyses

Total RNAs were extracted at the indicated times using the Nucleospin RNA XS kit (Macherey-Nagel). First-strand cDNAs were synthesized from 1 μg of total RNA by reverse transcription using the reverse transcriptase iScript system from Bio-Rad according to the manufacturer’s instructions. Quantitative RT-PCR was performed using a Bio-Rad CFX96 apparatus and qPCR Master Mix (Promega). Relative quantification of gene expression was normalized to the RPL13a mRNA expression level. Sequences of the PCR primers were as follows:

ALK1: F-CCTCACGAGATGAGCAGTCC, R- GGCGATGAAGCCTAGGATGTT;

ALK2: F-GTCATGGTTCAGGGAGACGG, R-CCAGAGTAGTGAGCTGAAGGT;

ALK3: F-ATGCAAGGATTCACCGAAAGC, R-AACAACAGGGGGCAGTGTAG;

BMPRII: F-TGGCAGTGAGGTCACTCAAG, R-TTGCGTTCATTCTGCATAGC;

ActRIIA: F-AGCAAGGGGAAGATTTGGTT, R-GGTGCCTCTTTTCTCTGCAC;

ActRIIB: F-CTGTGCGGACTCCTTTAAGC, R-TCTTCACAGCCACAAAGTCG;

BMP9: F-CAGATACACAACGGACAAATCGTC, R-TTGGCAGGAGACATAGAGTCGG;

BMP10: F- CCATGCCGTCTGCTAACATCATC, R-ACATCATGCGATCTCTCTGCACCA;

RPL13a: F- CCCTCCACCCTATGACAAGA, R- TTCTCCTCCAGAGTGGCTGT.

### Enzyme-linked immunosorbent assay (ELISA)

BMP9 and BMP10 ELISAs were performed with commercially available assays (R&D Systems). VEGF-A ELISA was performed with a commercially available assay (Mouse VEGF Quantikine ELISA Kit, R&D Systems).

### Cell proliferation, migration, viability and apoptosis

Cells were treated with 10 ng/mL of BMP9 or BMP10 in 0% FCS RPMI-1064 medium. Cell proliferation was assessed by counting cells every 24 h using an automated cell counter (TC20, Bio-Rad). Cell migration was assessed after wounding with a plastic pipette tip, placed back at 37 °C in the incubator and photographed at indicated times (0, 24, 48 and 72 h). Quantitation of monolayer closure was performed using Zen Blue software. Results are expressed as % of wound closure. Cell viability was assessed using the cell titer-Glo luminescent assay (Promega) at 24 h and 48 h after BMP9 addition. Cell apoptosis was assessed using the caspase-Glo 3/7 assay from Promega at 24 h and 48 h after BMP9 addition.

### Statistical analysis

Statistical data analysis was performed using the Mann-Whitney test except for tumor growth analysis, and in vitro proliferation and migration which were performed by the two-way Anova test using GraphPad Prism6.

## Results

### Loss of BMP9 increases tumor growth in the E0771 mammary carcinoma model

**Fig. 1:**
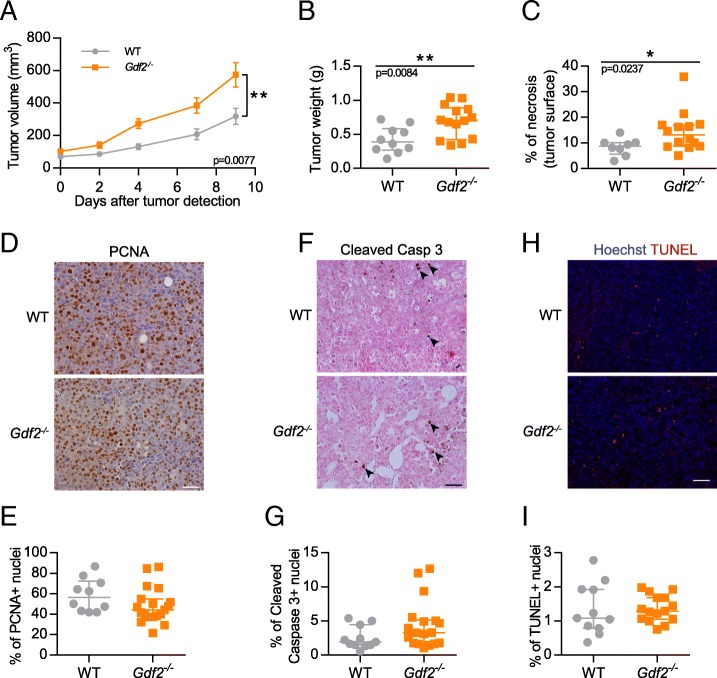
*Gdf2* deletion increases tumor growth in the E0771 mammary cancer model. E0771 cells were injected in the 4th mammary gland and tumor growth was assessed by caliper measurement every 2 to 3 days after tumor detection (**a**) and tumor weight measured (**b**) at the end of the experiment, 9 days after tumor detection (WT *n* = 10, *Gdf2*^−/−^ *n* = 14, 1 representative experiment out of 4). **c** Tumor necrosis area quantification (% of total tumor area) (WT *n* = 8, *Gdf2*^−/−^ n = 14, 2 experiments). **d**-**i** Representative images and quantitative analysis of the tumors stained for PCNA (**d**, **e**), cleaved-caspase 3 (black arrowheads **f**, **g**), (WT *n* = 11, *Gdf2*^−/−^ *n* = 18, 2 experiments). Scale bar 50 μm), and TUNEL (**h**, **i**) (WT n = 11, *Gdf2*^−/−^ *n* = 15, 1 experiment. Scale bar 100 μm) (**a**) Data are the mean ± SEM. Statistical analysis: Two-way matched ANOVA. (**b**, **c**, **e**, **g**, **i**) Data are the median ± interquartile range. Statistical analysis: Mann-Whitney test. *p ≤ 0.05 and **p ≤ 0.01 significantly different

To understand the contribution of BMP9 in tumor growth, we used the syngeneic orthotopic mouse mammary carcinoma models (E0771) [38]. The E0771 mammary cancer cell line is derived from a spontaneous adenocarcinoma recently described as claudin-low [39]. E0771 cancer cells were orthotopically injected into the fourth mammary gland of 6-week-old WT and *Gdf2-*deficient mice in the C57BL/6 background. We observed that *Gdf2*^*−/−*^ mice presented significantly larger and heavier tumors than WT mice (Fig. 1a and b). We next investigated whether these effects could be due to a direct effect of BMP9 on these cells. We first analyzed by quantitative RT-PCR the expression levels of the different type I and type II BMP receptors (Additional file 1: Fig. S1A and B). We found that both the mammary tumor cells (E0771) or the non-tumoral primary mammary epithelial cells (EpH4) expressed very low amount of ALK1, while, as expected, the endothelial cell line (H5V) expressed high levels of ALK1. All cells expressed ALK2 at a similar level, while ALK3 was expressed in E0771 and EpH4 cells but not in H5V cells. All cell types also expressed BMPRII and ActRIIA, whereas ActRIIB was below detectable level. None of these cells expressed BMP9 or BMP10 mRNA, which were also not detected in E0771 tumors (data not shown). In accordance with this receptor profile, we found that only high doses of BMP9 (5 ng/mL) induced Smad1/5/9 phosphorylation (Additional file 1: Fig. S1C), suggesting that this phosphorylation is not mediated by the high affinity receptor ALK1, in accordance with the low amount of the high affinity receptor ALK1 in these cells (Additional file 1: Fig. S1A), but by either ALK2 or ALK3. However, addition of high doses of BMP9 (10 ng/mL) did not affect E0771 cell growth, migration, viability or apoptosis (Additional file 1: Fig. S1D, E, F and G). In vivo, we also analyzed tumor necrosis and found that the percentage of necrosis was significantly higher in *Gdf2*^*−/−*^ mice than WT mice (Fig. 1c). Finally, we analyzed E0771 tumor sections to assess whether BMP9 affected tumor cell proliferation and apoptosis, in vivo, by counting the number of PCNA-, cleaved caspase 3- and TUNEL-positive cells and found no significant differences between WT and *Gdf2*^*−/−*^ mice (Fig. 1d, e, f, g, h and i).

### Loss of BMP9 decreases vessel perfusion in the mouse E0771 mammary carcinoma model

**Fig. 2:**
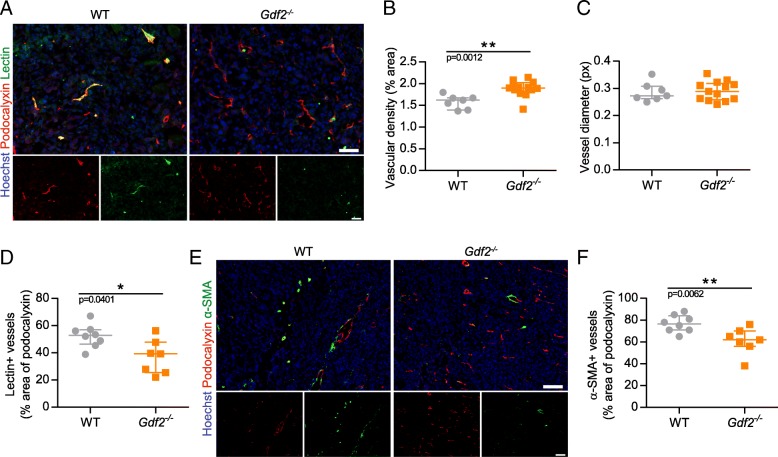
*Gdf2* deletion decreases tumor perfusion and maturation in the E0771 mammary cancer model. E0771 cells were injected in the 4th mammary gland of WT and *Gdf2*^−/−^ mice and tumor vascularization was analyzed 9 days after tumor detection. **a** Representative images of the tumors stained for podocalyxin (red), lectin (green) and cell nuclei (blue, Hoechst). Scale bar 50 μm. **b** Vascular density quantified by podocalyxin positive area (% of tumor area) and (**c**) assessment of vessel diameter using Ferret’s theorem (WT *n* = 7, *Gdf2*^−/−^ *n* = 13, 1 representative experiment out of 2). **d** Quantification of vessel perfusion by lectin staining (% area of lectin/podocalyxin) (WT n = 8, *Gdf2*^−/−^ n = 7, 1 representative experiment out of 3). **e** Representative images of the tumors stained for podocalyxin (red), α-smooth muscle actin (α-SMA) (green) and cell nuclei (blue, Hoechst). Scale bar 100 μm. **f** α-SMA staining quantification (% area of α-SMA/podocalyxin) (WT n = 8, *Gdf2*^−/−^ n = 7, 1 representative experiment out of 3). **b**, **c**, **d**, **f** Data are the median ± interquartile range. Statistical analysis: Mann-Whitney test. *p ≤ 0.05 and **p ≤ 0.01 significantly different

As we could not observe any effect of BMP9 on tumor cell proliferation that could explain the increase in tumor size in *Gdf2*^*−/−*^ mice, we next addressed whether the loss of BMP9 could be due to an effect on tumor angiogenesis. For this purpose, tumor sections were stained for the luminal endothelial cell marker podocalyxin [28, 30, 41], that we found to be the most suitable for automated vessel density quantification (this marker was validated by CD31 and von Willebrand Factor co-stainings, data not shown). Podocalyxin staining showed a slight but significant increase in vessel density in *Gdf2*^*−/−*^ mice versus WT mice (Fig. 2a and b) while there were no differences in vessel diameter (Fig. 2c), supporting that this increase in vessel density is due to an increase in vessel number rather than vessel size. VEGF-A being the main actor involved in tumor neovascularization, we analyzed circulating and tumor levels of VEGF-A by ELISA. We found no differences between *Gdf2*^−/−^ and WT mice (Additional file 2: Fig. S2A and B), supporting that the *Gdf2*^−/−^ phenotype is not directly linked to modifications of the VEGF-A pathway.

We next analyzed whether tumor vessels were functional by performing intravenous injection of FITC-conjugated tomato lectin that has a strong affinity for endothelial cells. We found that the percentage of tumor vessels perfused by lectin was significantly reduced in *Gdf2 *^−/−^ versus WT mice (Fig. 2a and d), supporting a defect in vessel perfusion in absence of BMP9. To test if this defect could be due to a lack of vessel maturation, we determined the presence of vessel-associated mural cells by performing immunostaining for α-smooth muscle actin (α-SMA). We found that the percentage of vessel coverage by α-SMA was also significantly reduced in *Gdf2*^*−/−*^ versus WT mice (Fig. 2e and f).

### Loss of BMP9 increases the number and the size of lung metastases in the mouse E0771 mammary carcinoma model

**Fig. 3:**
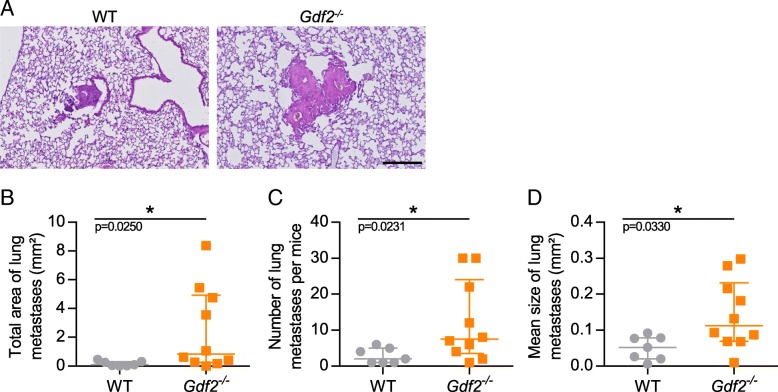
*Gdf2* deletion increases metastatic burden in the E0771 mammary cancer model. E0771 cells were injected in the 4th mammary gland of WT and *Gdf2*^−/−^ mice and lung metastases were analyzed 9 days after tumor detection. **a** Representative hematoxylin eosin colorations of metastases within WT and *Gdf2*^−/−^ lungs. Scale bar 250 μm. **b** Total area, (**c**) number and (**d**) mean size of lung metastases per mice bearing metastases (WT n = 7, *Gdf2*^−/−^ n = 10, 1 representative experiment out of 3). **b**, **c**, **d** Data are the median ± interquartile range. Statistical analysis: Mann-Whitney test. *p ≤ 0.05 significantly different

We next asked whether the loss of BMP9 could affect metastatic dissemination. The E0771 mammary cancer model has been shown to spontaneously disseminate in lungs [38]. We thus looked for lung metastases and found that the incidence of metastasis was similar in *Gdf2*^−/−^ compared to WT animal (72% vs 71%). However, the total area of lung metastases was significantly increased in *Gdf2*^*−/−*^ versus WT mice (Fig. 3a and b), which was linked to both an increase in the number and in the mean size of metastases (Fig. 3c and d). We next studied tumor lymphangiogenesis, an important route for lung metastases. Using LYVE-1 staining, we were able to detect lymphatic vessels in most of the E0771 tumors (respectively 61% and 72% of WT and *Gdf2*^*−/−*^ mice) but found no differences in lymphatic vessel density between *Gdf2*^*−/−*^ and WT mice (Additional file 3: Fig. S3A and B).

### Loss of BMP10 does not affect tumor growth, tumor angiogenesis and metastasis in the mouse E0771 mammary carcinoma model

**Fig. 4:**
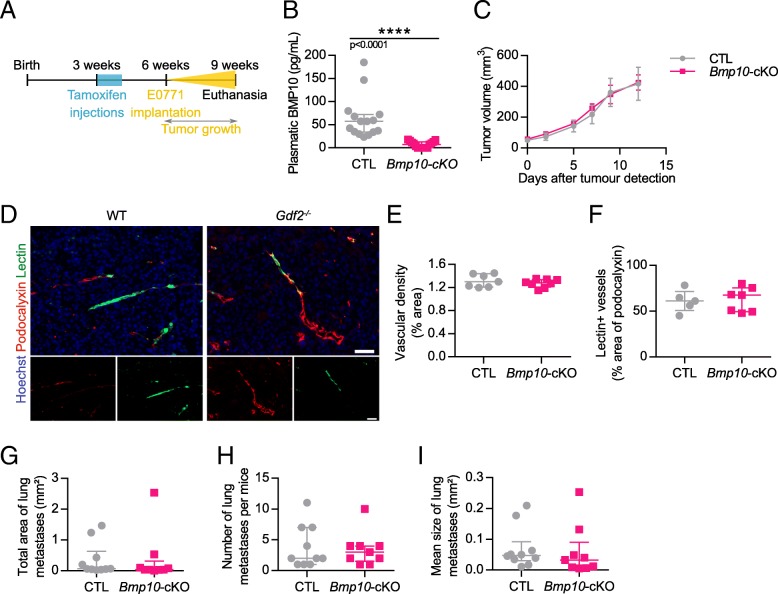
*Bmp10* conditional deletion has no impact on tumor growth, angiogenesis and lung metastasis in the E0771 mammary cancer model. **a** Schematic representation of the experimental protocol for *Bmp10* specific deletion and E0771 cells implantation. Tamoxifen was injected in all 3-week-old mice; 3 weeks later, E0771 cells were injected and tumor growth was analyzed for 3 weeks. **b** Plasmatic levels of BMP10 in control (CTL, n = 15) and *Bmp10* conditional KO (*Bmp10*-cKO, n = 15) mice assessed by ELISA at the end of the experiment. **c** Tumor growth was assessed by caliper measurement every 2 to 3 days after tumor detection (CTL n = 7, *Bmp10*-cKO n = 8, 1 representative experiment out of 3). **d** Representative images of the tumors stained for podocalyxin (red), lectin (green) and cell nuclei (blue, Hoechst). Scale bar 50 μm. **e** Vascular density quantified by podocalyxin surface area (% of tumor area) and (**f**) Quantification of vessel perfusion by lectin staining (% area of lectin/podocalyxin) (CTL n = 7, *Bmp10*-cKO n = 8, 1 representative experiment out of 3). **g** Total area, (**h**) number and (**i**) mean size of lung metastases per mice bearing metastases (CTL n = 10, *Bmp10*-cKO *n* = 9, 2 experiments). **c** Data are the mean ± SEM. Statistical analysis: Two-way matched ANOVA. **b**, **e**, **f**, **g**, **h**, **i** Data are the median ± interquartile range. Statistical analysis: Mann-Whitney test. ****p ≤ 0.001 significantly different

We next addressed the role of BMP10, the other ALK1’s ligand, in the E0771 mammary cancer model. As *Bmp10* deletion is embryonic lethal due to cardiac defects [21], we generated a conditional Rosa26 CreER^T2^;*Bmp10*^lox/lox^ mice that allowed deletion of *Bmp10* after tamoxifen injection. Both Cre-positive (*Bmp10*-cKO) and Cre-negative (CTL) mice were injected with tamoxifen when three-week-old (Fig. 4a). At the age of six weeks, E0771 cells were injected into the fourth mammary gland. On the day of euthanasia, blood was collected and circulating BMP10 levels were measured by ELISA. As expected, tamoxifen treatment significantly decreased BMP10 circulating levels (64 and 7 pg/mL, in CTL and *Bmp10-*cKO mice, respectively, Fig. 4b). We found that loss of BMP10 did not significantly modify tumor volume (Fig. 4c). We next analyzed tumor vascularization but could not detect any differences in tumor vessel density (podocalyxin staining), nor in vessel perfusion (lectin injection) between CTL and *Bmp10*-deleted mice (Fig. 4d-f). We also studied lung metastasis in these mice and could not detect any differences in the total area, number or mean size of lung metastases between CTL and *Bmp10*-cKO mice (Fig. 4g-i).

We tested whether this absence of effect in the *Bmp10*-cKO mice could be due to a compensation by BMP9. However, we found similar levels of BMP9 mRNA in the liver of CTL and *Bmp10-*cKO mice (Additional file 4: Fig. S4).

### The loss of BMP9 and BMP10 in double knockout mice does not lead to a stronger effect on mouse E0771 mammary carcinoma tumor growth and angiogenesis than the single loss of BMP9

**Fig. 5:**
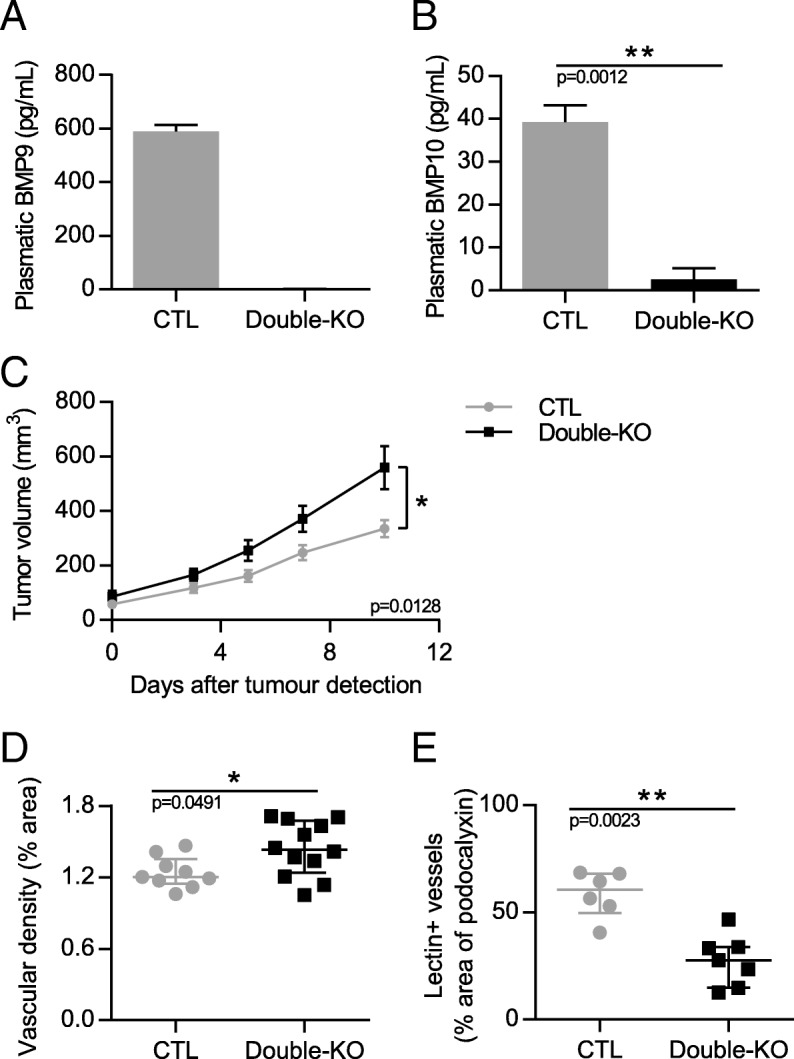
Double deletion of *Gdf2* and *Bmp10* increases tumor growth and decreases tumor perfusion in the E0771 mammary cancer model. Tamoxifen was injected in all 3-week-old mice; 3 weeks later E0771 cells were injected and tumor growth was analyzed for 3 weeks. **a**, **b** BMP9 and BMP10 circulating levels in CTL and double-KO mice assessed by ELISAs at the end of the experiment. **c** Tumor growth was assessed by caliper measurement every 2 to 3 days after tumor detection (CTL n = 13, Double-KO *n* = 11, 2 experiments). **d** Vascular density quantified by podocalyxin surface area (% of tumor area) (CTL n = 9, Double-KO *n* = 12, 1 representative group out of 4) and (**e**) Quantification of vessel perfusion by lectin staining (% area of lectin/podocalyxin) (CTL *n* = 6, Double-KO n = 7, 1 representative group out of 4). **a**, **b** Data are the mean ± SEM. Statistical analysis: Mann-Whitney. **c** Data are the mean ± SEM. Statistical analysis: Two-way matched ANOVA. **d**, **e** Data are the median ± interquartile range. Statistical analysis: Mann-Whitney test. *p ≤ 0.05, and **p ≤ 0.01 significantly different

We next addressed whether the loss of both BMP9 and BMP10 within the same mice would have a more pronounced effect on the E0771 mammary cancer model. These mice were generated by crossing *Gdf2*^*−/−*^mice with Rosa26 CreER^T2^;*Bmp10*^lox/lox^ mice. Both Cre-positive (*Gdf2*^*−/−*^;*Bmp10*^lox/lox^, referred as double-cKO) and Cre-negative (*Gdf2*^*+/+*^;*Bmp10*^lox/lox^, referred as CTL) mice were injected with tamoxifen at the age of three weeks. As expected, the circulating levels of BMP9 and BMP10 measured after euthanasia by ELISA were strongly reduced in double-KO mice as compared to CTL mice (Fig. 5a and b). These double-KO mice were viable during the eight weeks of the experimental procedure. At the age of six weeks, E0771 cells were injected as described in Fig. 4a. We found that double-KO mice developed larger tumors than CTL mice (Fig. 5c). They also presented a slight increase in vessel density and a decrease in perfused vessels (Fig. 5d and e). Together these data showed that the double-KO mice, in this experimental model of E0771 mammary cancer, behaved as *Gdf2*^*−/−*^*mice.*

## Discussion

Several clinical trials targeting ALK1 or its co-receptor endoglin are ongoing although the role of these two receptors in a tumor context are not yet understood and, so far present limited beneficial results [25]. Since both ALK1’s ligands, BMP9 and BMP10 circulate in blood, there is an urgent need to understand the respective contribution of each ligand in tumor development and metastasis. Contrary to what we expected from many preclinical studies aiming at blocking ALK1, we found that loss of BMP9 led to an increase in tumor growth, combined with decreased tumor vessel maturation and increased lung metastasis in the E0771 model. On the other hand, loss of BMP10 did not seem to affect the E0771 mammary cancer model and the double deletion of BMP9 and BMP10 did not lead to a stronger phenotype than the single deletion of BMP9. Together these results demonstrate, for the first time, that BMP9 and BMP10 exhibit distinct roles in tumor growth, angiogenesis and metastatic dissemination.

We show that the loss of BMP9 led to a small but statistically significant increase in tumor volume. However, this result does not seem to be a consequence of a direct effect of BMP9 on tumor cell proliferation since BMP9 did not affect E0771 cell proliferation, cell viability nor apoptosis in vitro*.* In accordance, we could not observe any significant differences in PCNA staining nor on activated caspase-3 or TUNEL stainings on tumors that were harvested at the end of the study. This is in contrast to other studies that have shown that overexpression of BMP9 using adenoviruses inhibit the growth, invasion and migration of the breast cancer cell lines MDA-MB-231, SK-BR-3 and 4 T1 [35, 42, 43]. This could be due to differences in breast cancer models or in the doses of BMP9 used. On the other hand, we found that loss of BMP9 affected tumor neovascularization. Indeed, we found that the loss of BMP9 increased tumor vessel density, and decreased tumor vessel perfusion and coverage by mural cells as illustrated by α-SMA staining, indicating decreased vessel maturation. This is in accordance with the current hypothesis that BMP9 is a maturation or “normalization” factor [44]. The tumor vasculature has been described as a dense but chaotic and heterogeneous network of structurally and functionally abnormal vessels with a compromised blood flow, a lack of pericyte coverage and a high permeability with non-specific extravasation of blood components [45]. “Normalization” of the tumor vasculature through the Angiopoietin-Tie2 axis, for example, decreases vessel density, increases vessel coverage and perfusion while decreasing permeability. As a consequence of this vessel normalization, tumor growth is decreased as well as tumor necrosis and metastasis [46]. Our results on E0771 tumor growth, necrosis, metastasis and tumor perfusion are in accordance with this concept. Together, our data support that BMP9 is a circulating vascular quiescence factor in both physiological and tumoral contexts.

Most cancer patients die of their metastases rather than of their primary tumor so therapeutic strategies have recently focused on metastasis. In the E0771 model, we found that *Gdf2*^*−/−*^ mice developed more and larger metastases. This supports the normalization theory where heterogeneity, leakage and lack of perfusion leads to hypoxia and aggravates tumor progression and metastasis (which is hindered upon normalization) [47]. It is also possible that, as for the primary tumor, the growth of the metastases would be favored in *Gdf2*^−/−^ mice. Differences in lymphangiogenesis could also be an explanation as we have previously shown that *Gdf2* deletion in the C57BL/6 background leads to lymphatic drainage deficiency [22]. However, although we detected lymphatic vessels in these tumors, we found no differences in tumor lymphatic vessel density between WT and *Gdf2*^*−/−*^ mice.

It was recently shown, in another preclinical study, that blocking BMP9, using a neutralizing anti-BMP9 antibody, significantly reduced renal tumor growth and reduced tumor vascular permeability [48] suggesting potential differences between different tumor types. The role of BMP9, using a similar approach of *Gdf2* knockdown, has recently been described in the pancreatic RIP1-TAg2 PanNETS cancer model [49]. However, in this model, *Bmp9* ablation led to a reduced tumor volume, no effect in vessel density but an increase in vessel branching and pericyte coverage and an increase in metastasis. These results, apart for the increase in metastasis, differ from our results. This might be due to differences between cancer types. However, in this paper, using the same pancreatic model, the authors obtained different and even opposite results with mice deleted within this signaling pathway (*Eng*^*+/−*^*, Acvrl1*^*+/−*^ and *Gdf2*^*−/−*^ mice) [49], highlighting the difficulties of understanding the role of this pathway in cancer and tumor angiogenesis. Nevertheless, their results [49] and ours show that, in these two cancers, the loss of BMP9 leads to an increase in metastasis and thus cautions against blockade of this BMP9/ALK1 pathway in cancer treatments.

We found that, in contrast to the loss of BMP9, the loss of BMP10 had no significant effect in the E0771 mammary carcinoma model and the loss of both BMP9 and BMP10 in the double-Knockout mice did not lead to a stronger phenotype than the single loss of BMP9. This was not due to tamoxifen injection as *Gdf2*-KO mice injected with tamoxifen also showed significantly increased tumor growth (data not shown). Tamoxifen injection led to a 90% decrease in BMP10 circulating levels. It is unlikely, that the remaining 10% of BMP10 could explain the lack of effect. Our results rather support that BMP10 does not play an important role in this breast tumor model. It is interesting to note that loss of BMP9 is sufficient to affect tumor development in this mammary carcinoma model. This supports that, in contrast to post-natal model of angiogenesis or vascular remodeling [17, 20, 23], there is no redundancy between BMP9 and BMP10 in this tumor context. This absence of redundancy could be due to differences between physiological and tumor angiogenesis or due to the fact that the role of BMP9 and BMP10 might be different in newborns and adults. Indeed, we have previously shown that blocking BMP10 with a neutralizing antibody in newborn *Gdf2*^*−/−*^ mice led to the death of these pups within few days [23], which is not the case here in adult mice.

## Conclusions

There are currently several anticancer drugs targeting ALK1 or its ligands in phase I/II of clinical development although with limited beneficial results so far [5, 25]. Our present work highlights the need of performing detailed mechanistic studies prior to pursuing clinical testing of drugs impinging this pathway. Our studies show that targeting BMP9 or BMP10 differently affect tumor growth, angiogenesis and metastatic dissemination. These data also support that targeting specifically BMP9 rather than ALK1 or endoglin, that will affect both ligands, could be more appropriate. It also addresses the question whether blocking this pathway is relevant in cancer treatments as, at least in this mammary mouse model, loss of BMP9 increases tumor growth and lung metastasis. Anti-angiogenic treatments have slightly changed optic recently and it is not clear whether drugs should block angiogenesis, sustain vessel normalization or promote vascularization [50]. Thus, BMP9 might be an interesting quiescence factor in the context of vessel normalization but in this case, we might want to activate this pathway by giving recombinant BMP9 in cancer than rather blocking it and to combine it with current chemotherapies or immunotherapies.

## Additional files

Additional file 1: **Figure S1.** Characterization of E0771 cells in vitro and their response to BMP9. (PDF 679 kb)

Additional file 2: **Figure S2.** VEGF-A levels in the E0771 mammary cancer model. (PDF 78 kb)

Additional file 3: **Figure S3.** *Gdf2* deletion has no impact on tumor lymphangiogenesis in the E0771 breast cancer model. (PDF 1463 kb)

Additional file 4: **Figure S4.** BMP9 mRNA levels in liver of *Bmp10*-cKO mice. (PDF 57 kb)

## Abbreviations

ActR2: Activin type 2 receptor
ALK1: Activin receptor-like kinase 1
BMP: Bone morphogenetic protein
BMPR2: BMP type 2 receptor
GDF: Growth and differentiation factor
PCNA: proliferating cell nuclear antigen
SMA: Smooth muscle actin
VEGF: Vascular endothelial growth factor
WT: Wild type
