# Supplementary figures and images for: BMP9, but not BMP10, acts as a quiescence factor on tumor growth, vessel normalization and metastasis in a mouse model of breast cancer
Source: J Exp Clin Cancer Res. 2018 Aug 30;37:209. doi: 10.1186/s13046-018-0885-1 (PMC6118004; doi:10.1186/s13046-018-0885-1)

**Figure S1****A**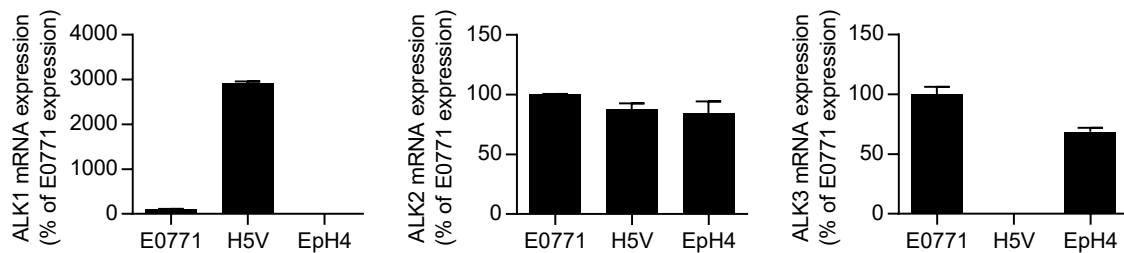**B**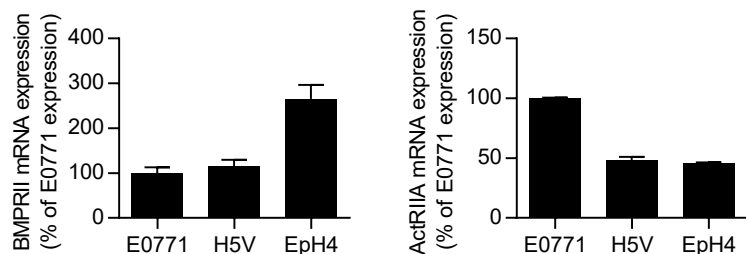**C**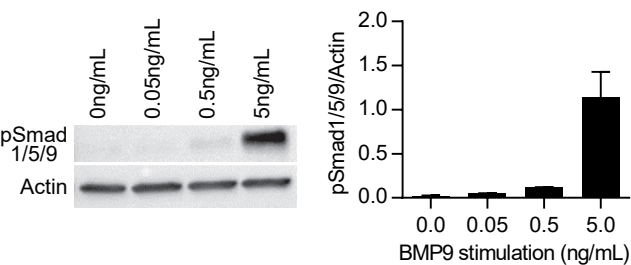**D**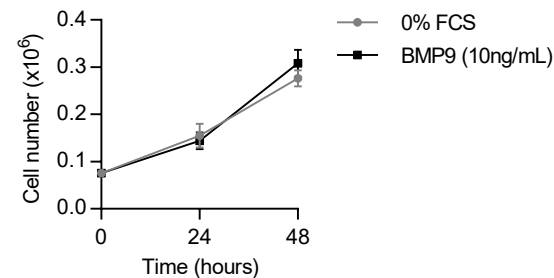**E**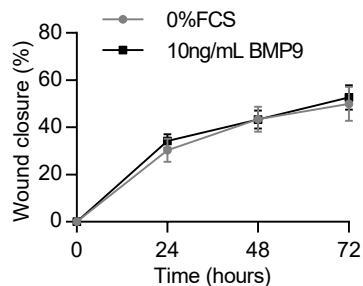**F**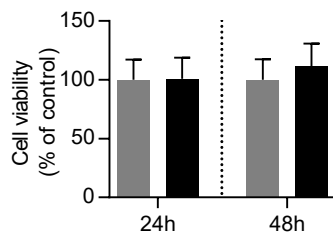**G**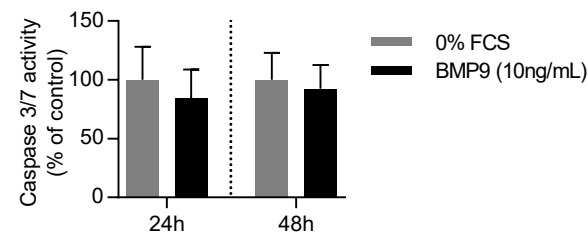

Supplement: Supplementary file 1 — Figure S1. Characterization of E0771 cells in vitro and their response to BMP9. (PDF 679 kb) [file 13046_2018_885_MOESM1_ESM.pdf]

Figure S2

A

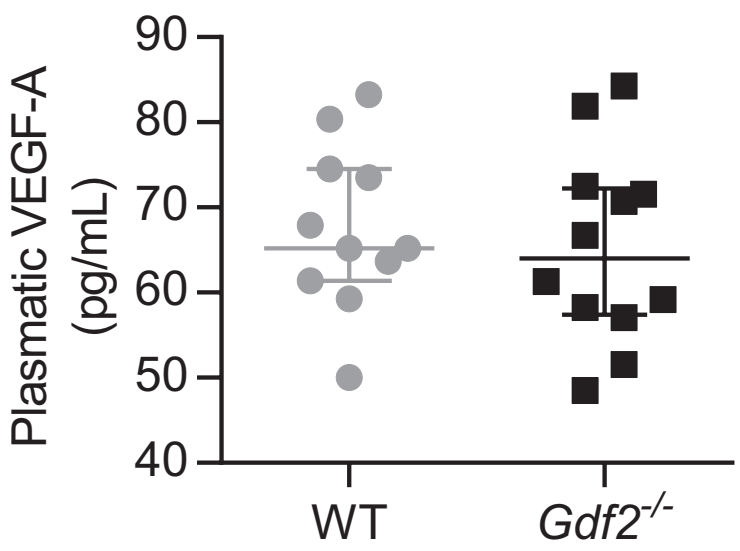

B

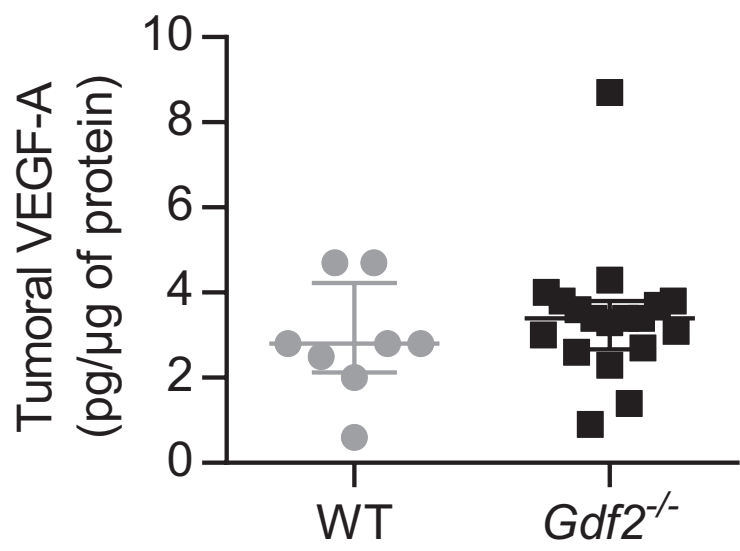

Supplement: Supplementary file 2 — Figure S2. VEGF-A levels in the E0771 mammary cancer model. (PDF 78 kb) [file 13046_2018_885_MOESM2_ESM.pdf]

**Figure S3**

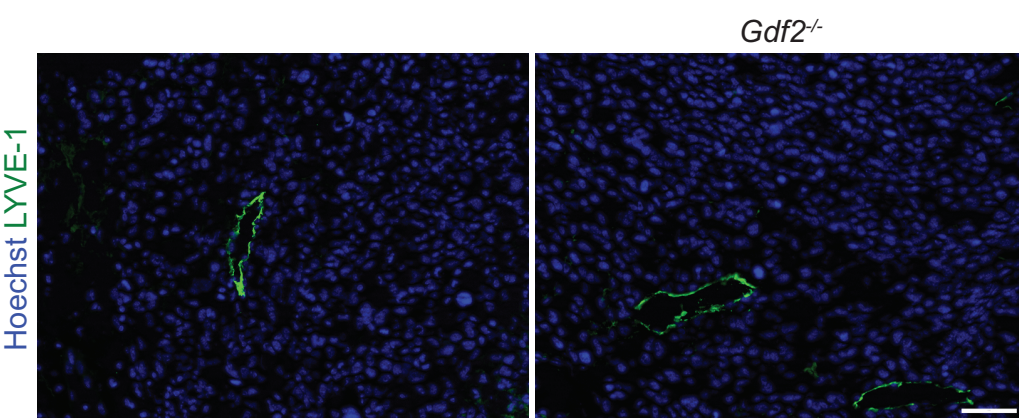

**B**

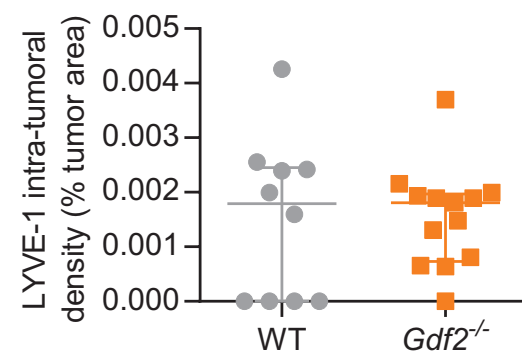

Supplement: Supplementary file 3 — Figure S3. Gdf2 deletion has no impact on tumor lymphangiogenesis in the E0771 breast cancer model. (PDF 1463 kb) [file 13046_2018_885_MOESM3_ESM.pdf]

# Figure S4

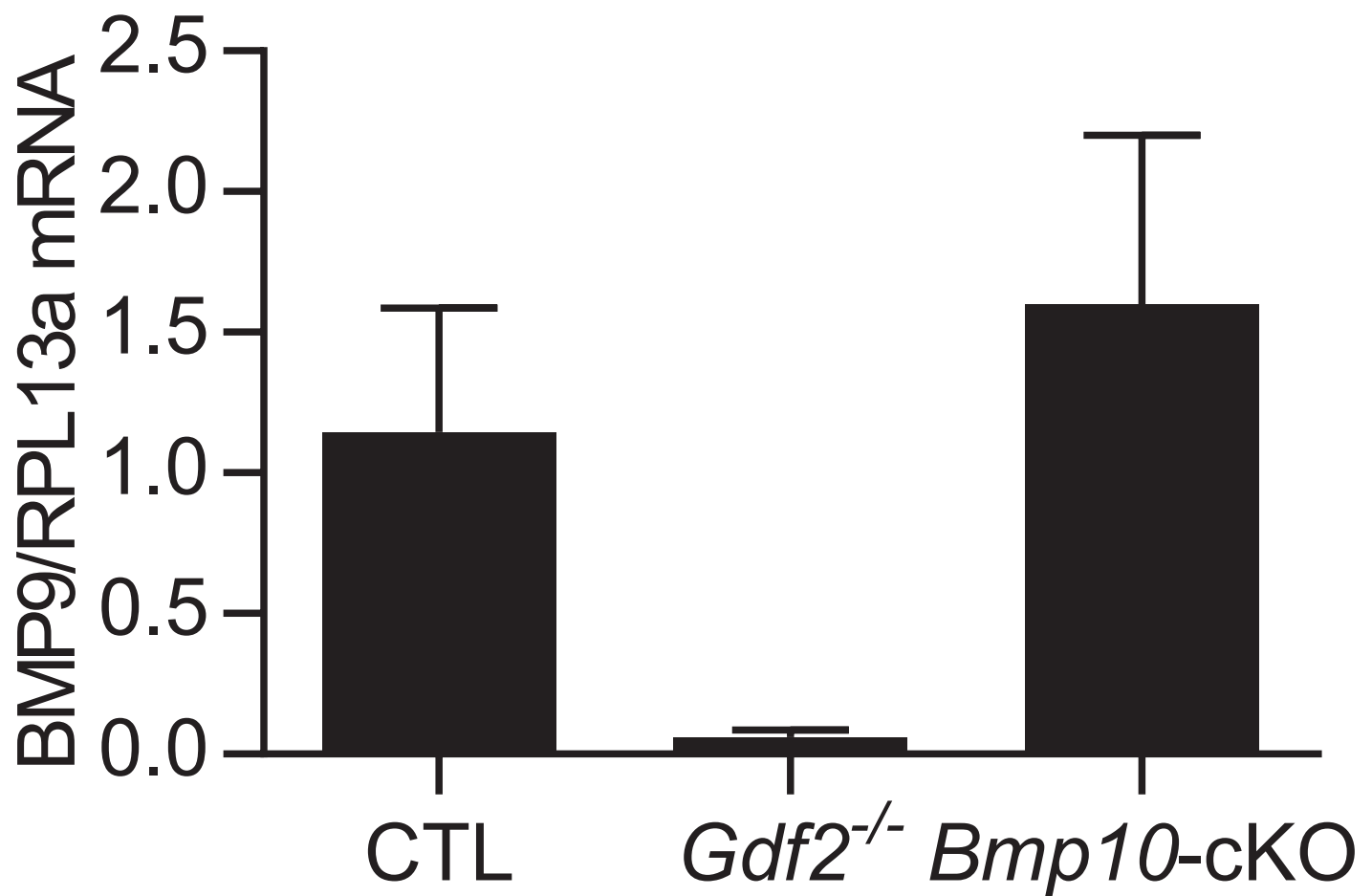

Supplement: Supplementary file 4 — Figure S4. BMP9 mRNA levels in liver of Bmp10-cKO mice. (PDF 57 kb) [file 13046_2018_885_MOESM4_ESM.pdf]
